# Correction: Dissecting contact tracing apps in the Android platform

**DOI:** 10.1371/journal.pone.0258074

**Published:** 2021-09-27

**Authors:** Vasileios Kouliaridis, Georgios Kambourakis, Efstratios Chatzoglou, Dimitrios Geneiatakis, Hua Wang

There is an error in the second author’s affiliation. The correct affiliation is: European Commission, Joint Research Centre (JRC), 21027 Ispra (VA), Italy.
